# Machine learning for screening prioritization in systematic reviews: comparative performance of Abstrackr and EPPI-Reviewer

**DOI:** 10.1186/s13643-020-01324-7

**Published:** 2020-04-02

**Authors:** Amy Y. Tsou, Jonathan R. Treadwell, Eileen Erinoff, Karen Schoelles

**Affiliations:** Center for Clinical Excellence and Guidelines, ECRI Institute, Evidence-based Practice Center, 5200, Plymouth Meeting, PA 19462-1298 USA

**Keywords:** Machine learning, Citation screening, Text-mining, Abstrackr, EPPI-Reviewer, Screening prioritization, Methodology, Screening burden, Efficiency

## Abstract

**Background:**

Improving the speed of systematic review (SR) development is key to supporting evidence-based medicine. Machine learning tools which semi-automate citation screening might improve efficiency. Few studies have assessed use of screening prioritization functionality or compared two tools head to head. In this project, we compared performance of two machine-learning tools for potential use in citation screening.

**Methods:**

Using 9 evidence reports previously completed by the ECRI Institute Evidence-based Practice Center team, we compared performance of Abstrackr and EPPI-Reviewer, two off-the-shelf citations screening tools, for identifying relevant citations. Screening prioritization functionality was tested for 3 large reports and 6 small reports on a range of clinical topics. Large report topics were imaging for pancreatic cancer, indoor allergen reduction, and inguinal hernia repair. We trained Abstrackr and EPPI-Reviewer and screened all citations in 10% increments. In Task 1, we inputted whether an abstract was ordered for full-text screening; in Task 2, we inputted whether an abstract was included in the final report. For both tasks, screening continued until all studies ordered and included for the actual reports were identified. We assessed potential reductions in hypothetical screening burden (proportion of citations screened to identify all included studies) offered by each tool for all 9 reports.

**Results:**

For the 3 large reports, both EPPI-Reviewer and Abstrackr performed well with potential reductions in screening burden of 4 to 49% (Abstrackr) and 9 to 60% (EPPI-Reviewer). Both tools had markedly poorer performance for 1 large report (inguinal hernia), possibly due to its heterogeneous key questions. Based on McNemar’s test for paired proportions in the 3 large reports, EPPI-Reviewer outperformed Abstrackr for identifying articles ordered for full-text review, but Abstrackr performed better in 2 of 3 reports for identifying articles included in the final report. For small reports, both tools provided benefits but EPPI-Reviewer generally outperformed Abstrackr in both tasks, although these results were often not statistically significant.

**Conclusions:**

Abstrackr and EPPI-Reviewer performed well, but prioritization accuracy varied greatly across reports. Our work suggests screening prioritization functionality is a promising modality offering efficiency gains without giving up human involvement in the screening process.

## Background

Systematic reviews (SRs) play a vital role across many disciplines including education, criminal justice, and particularly healthcare, enabling evidence-based medicine by synthesizing and critically appraising bodies of literature to produce a comprehensive and critical assessment of what is known [1, 2]. In particular, SRs impact patient care as the basis for development of high-quality clinical practice guidelines [3, 4]. Despite their importance, creating SRs in a timely fashion remains challenging due to the lengthy process required for development, which typically requires months to years [5].

One particularly time-consuming step in developing SRs is citation screening. Typically, thousands of abstracts are reviewed to identify only a small number of relevant studies. While laborious, this process reflects the priority systematic reviewers place on identifying *all* relevant studies to avoid bias (i.e., attaining 100% sensitivity) [6]. Wallace et al. estimated that for an experienced reviewer, screening 5000 abstracts requires 40 h of uninterrupted work for simple topics, with significantly more time for complex topics [7]. In one project at our institution consisting of five large SRs (for use in guideline development), searches identified a combined 31,608 citations, for which we screened 13,492 abstracts in 297 h, or 7.4 full weeks for one investigator.

**Fig. 1:**
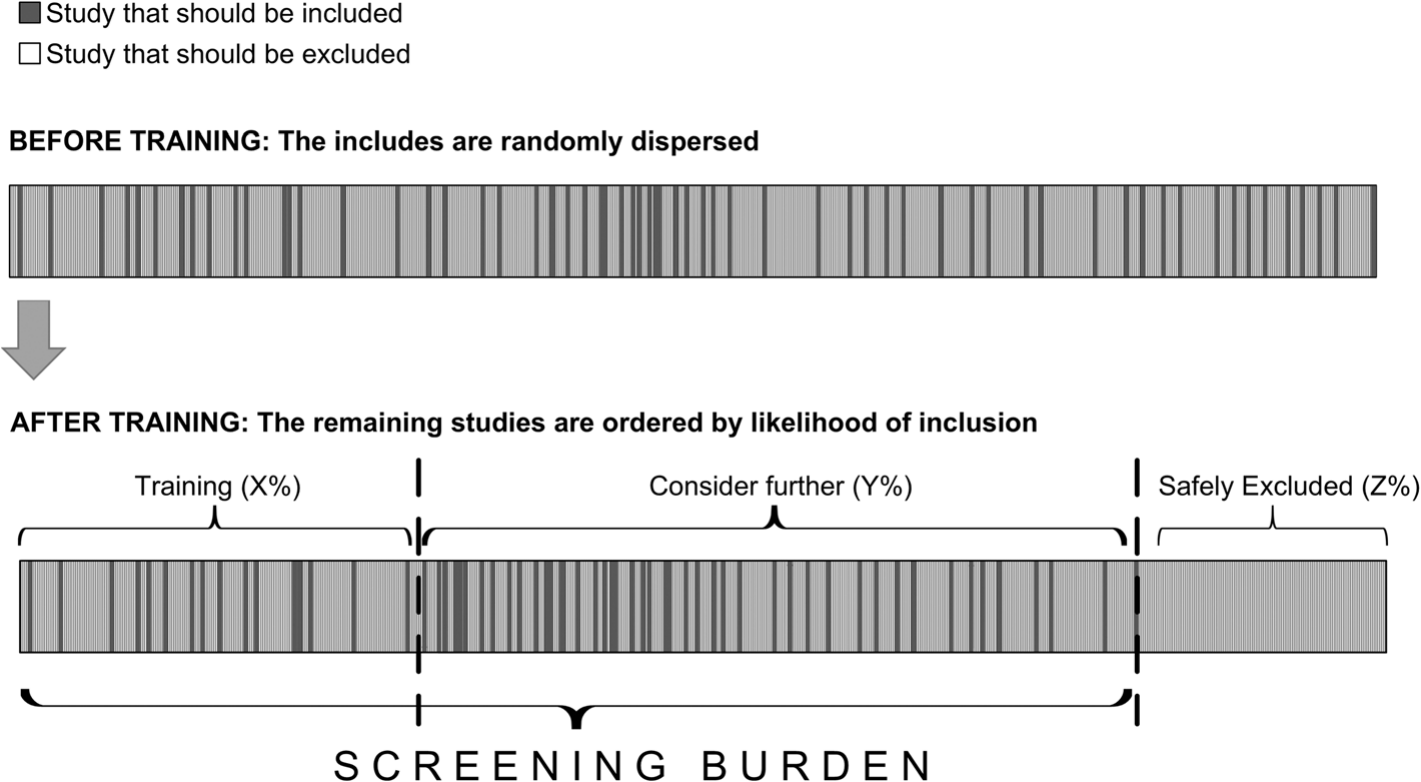
Screening prioritization and potential reduced screening burden. This figure demonstrates graphically how screening prioritization works. Prior to screening, the articles ultimately included are randomly dispersed (top half). Reviewers train the algorithm by manually including/excluding studies until a pre-specified number of studies are included. The algorithm then generalizes rules and prioritizes the remaining studies for evaluation by the reviewer

Semi-automating this process using innovative citation screening tools represents one potential solution [8, 9]. Using machine-learning algorithms trained on a subset of citations, these tools order citations for screening, presenting abstracts with the highest probability of meeting inclusion criteria first (see Fig. 1). This process, known as screening prioritization does not directly reduce the number of abstracts which require screening. However, by presenting reviewers with citations more likely to be included, screening prioritization can improve efficiency by promoting early identification of relevant citations, accelerating retrieval of full-text articles and data extraction and workflow planning [10, 11].

Screening prioritization can also support another strategy for improving efficiency, screening truncation, in which citations that fall beneath a specified probability for inclusion are automatically excluded (e.g., < 0.1%). This functionality is sometimes described as automatic classification, since the machine “classifies” the remaining unscreened abstracts as so unlikely to be relevant that they do not require screening and can simply be designated as “excluded” [10]. Thus, unlike screening prioritization, screening truncation can allow reviewers to screen fewer abstracts.

Together, screening prioritization and screening truncation can decrease *screening burden*, which may be defined as the proportion of citations screened in order to identify all relevant studies. Citation screening tools utilizing these functionalities have the potential to reduce workload by 30 to 70% (with estimates as high as 90%) [12–14]. However, because truncating the screening process risks missing important studies, this strategy has not been widely adopted [10]. Assessing the accuracy of screening prioritization (to determine to what extent tools can actually identify relevant citations earlier) could help alleviate this concern by identifying various thresholds at which screening truncation may be “safely” performed to avoid missing any relevant studies.

A 2016 AHRQ Evidence-based practice center (EPC) report surveying the landscape of text-mining tools for use in SR development noted several research priorities including (1) further validation of workload reductions, (2) characterization of potential performance differences for large vs. small datasets, and (3) direct comparisons of tools [15]. To address these research gaps, in this study, we assessed the efficacy of 2 off-the-shelf citation screening tools (Abstrackr and EPPI-Reviewer) to reduce screening burden using screening prioritization. In addition, we also sought to determine if reductions in screening burden differed for large compared to small evidence reports.

### Tools of interest: Abstrackr and EPPI-Reviewer

Abstrackr and EPPI-Reviewer are two off-the-shelf screening tools designed by established systematic review groups. Abstrackr [16, 17] is an open source application currently maintained by the Brown Center for Evidence Synthesis in Health; EPPI-Reviewer [18] was developed at the University College London and is currently used to support evidence synthesis for organizations including the Cochrane Review Groups [14]. Both tools are web-based and facilitate citation screening through text-mining tools and machine learning techniques; they employ “active learning,” in which ongoing feedback from reviewers is used dynamically to improve predictive accuracy [14, 17]. Several studies have incorporated Abstrackr into workflow (e.g., as a second screener) and reported a range of improvements in efficiency [19]. For instance, three studies using Abstrackr for screening citations for new reviews (i.e., not updates) reported significant, but variable workload reductions ranging from 9 to 57% [7, 13, 17]. However, to our knowledge, no studies have assessed EPPI-Reviewer.

## Methods

### Study design

We compared the accuracy of screening prioritization by Abstrackr vs. EPPI-Reviewer using citations from a sample of 9 previously completed evidence reports. We selected 3 evidence reports which had each required screening a large number of citations along with 6 reports which had required screening for a smaller number of citations. Throughout the rest of this report, we describe these reports as “large” or “small” evidence reports.

To be considered for selection, evidence reviews had to assess well-defined medical interventions, be performed since 2010, and screen a minimum number of citations (> 1000 citations for large reports, 200–999 citations for small). For large reviews, we selected 3 AHRQ Evidence-based Practice Center (EPC) reports published from 2012 to 2018; as typical for EPC reports, we performed dual screening. For smaller reports, we selected most from “Emerging Technology” reports ECRI Institute produces in response to requests from health systems regarding specific interventions. These reports employ standard systematic review methodology (comprehensive search, screening, risk of bias assessment, and strength of evidence appraisal); however, citation screening is performed by only a single analyst.

### Citation screening process

**Fig. 2:**
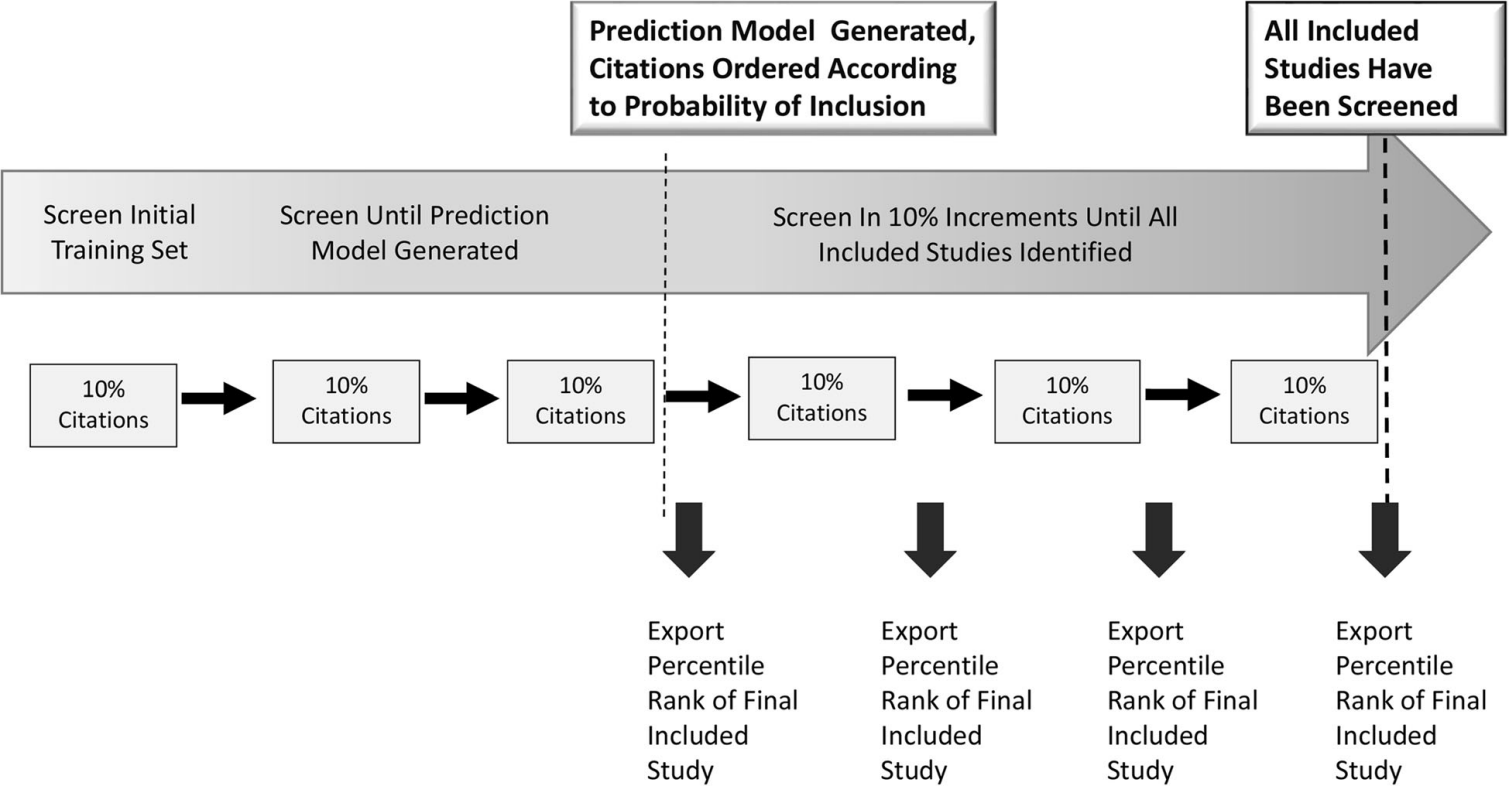
Citation screening process. This figure shows our process for analyzing ordered lists in 10% increments. During training, each tool received sufficient input to generate a prediction algorithm and prioritize all remaining unscreened citations by ordering them from most to least relevant. After this point, we exported ordered lists of unscreened remaining abstracts at each 10% increment (for each evidence report) until all relevant articles had been presented for screening by each tool

The citation screening process we used for this project is summarized in Fig. 2. While both tools support screening prioritization, there are key differences. Abstrackr updates prediction algorithms once a day (overnight). Conversely, for EPPI-Reviewer, the prediction algorithm updates iteratively (e.g., every 25 citations). With this difference in mind, for each evidence report, we trained each tool using the same sets of citations but different approaches: for Abstrackr, we used a random 10% sample of all citations; for EPPI-Reviewer, we screened 10 random citations and then engaged the priority screening mode until 10% of total citations had been screened. Screening continued in 10% increments. After generation of a prediction model, each tool presented citations in order from highest to lowest likelihood of meeting inclusion criteria.

Systematic reviews may be performed de novo or serve to update a prior existing review. Thus, for each evidence report, we characterized screening burden in two ways (see Table 1). In Task 1, to mimic workflow for a new review, we compared screening performance to decisions made by human reviewers (e.g., should this abstract be included for *full-text review*). In this scenario, the algorithm could potentially be misled by reviewers choosing to include studies which are ultimately excluded. However, for a review update, an algorithm could be trained solely using known included studies from a prior review (which could dramatically improve accuracy of screening prioritization). To approximate this scenario, in Task 2, we considered whether accuracy of predictions improved when tools were trained using citations ultimately included in the final report.

**Table 1:** Task 1 and 2: inputs and outcomes

	Input to Abstrackr/EPPI-Reviewer	Outcome (prioritization accuracy) definition
**Task 1**	Did human reviewer(s) order the study for full-text review?	Proportion of citations screened to reach all citations ordered for full-text review
**Task 2**	Was the citation included in the final report?	Proportion of citations screened to reach all citations included in the final report

We exported ordered lists at each 10% interval (including the percentile rank of the final ordered/included study). Although Abstrackr does provide numerical probabilities of inclusion, we only used the ordered lists of citations to enable comparisons across both tools (as EPPI-Reviewer does not provide probabilities). Screening continued until *all ordered/included studies* had been screened*.* For small reports (with fewer citations), we anticipated that Abstrackr or EPPI-Reviewer might have insufficient information to generate a prediction model; in those cases, we planned to continue screening in 10% increments until all citations had been screened.

### Outcome measures

Our primary outcome measures were prioritization accuracy and the proportion of included studies identified at various thresholds.

#### Prioritization accuracy

For each evidence report, we used the percentile rank of the final included study to determine prioritization accuracy/based on exported predictions, we determined whether each excluded abstract fell within the “screening burden,” (the proportion of citations which require screening in order to detect all included studies) for EPPI-Reviewer and Abstrackr respectively. Any particular excluded abstract might fall into the screening burden for *neither* or *both* EPPI-Reviewer and Abstrackr; alternatively, excluded abstracts might fall in one tool’s screening burden but not the other’s. Thus, the data are paired proportions, and we assessed the effect using odds ratios and McNemar’s test [20].

For statistical power, the power of McNemar’s test is 73.5% for a small report (400 abstracts total) for which 10% of abstracts fell into the screening burden of Abstrackr but not EPPI-Reviewer, and another 5% of abstracts fell into the screening burden of EPPI-Reviewer but not Abstrackr.

#### Sensitivity at various thresholds

Thresholds for screening truncation may be determined in several ways: screening may be stopped below a particular probability of inclusion or percentile rank or after consecutive exclusion of a large number of citations (e.g., inclusion rate < 0.1% in the last 500 screened). For each 10% set (i.e., 10%, then 20%, and then 30%), we calculated the hypothetical sensitivity of the tool, which was the percentage of eventually included articles that had been screened by the tool at that point. This provided data on the time course of machine learning, which may vary by tool (e.g., Abstrackr or EPPI-Reviewer) or review (e.g., pancreatic cancer imaging or inguinal hernia treatment).

## Results

### Included evidence reports and preparation of training sets

Our databases contained 19,149 citations identified by searches for possible inclusion in these 9 reports. We excluded 505 duplicates to avoid delivering “mixed messages” for the machine learning algorithms (i.e., if a citation was included, but its duplicate was excluded for being a duplicate, this would confuse algorithms trained on abstract content alone). As we were unsure if either algorithm incorporated publication year into its prioritization scheme, we excluded 363 citations which had been excluded from the original pancreatic cancer imaging review solely for being published prior to 2000. We included the remaining 18,281 articles.

Included reports covered a range of clinical topics, literature base sizes, and proportion of full-text orders and studies ultimately included (see Table 2). Although 10 articles appeared in searches for multiple topics, this did not impact our analytic comparisons, as citations from each report were entered as independent projects within each citation screening tool. In the two rightmost columns of Table 2, we provide the total number of articles that were ultimately ordered as full articles by the original reviewers (Task 1) as well as the total number of articles that were ultimately included in the review by the original reviewers (Task 2).

**Table 2:** Citation disposition for 9 completed reports

Report	Citations for screening	Size of each 10% training set	Citations originally selected for full text screening (***n***, %)	Citations included in final report (***n***, %)
***Large reports***				
Pancreatic cancer imaging	9038	904	696 (8%)	104 (1%)
Indoor allergen reduction	3181	318	200 (6%)	72 (2%)
Surgical procedures for inguinal hernia	2706	271	843 (31%)	223 (8%)
***Small reports***				
Dabigatran	889	89	107 (12%)	4 (0.5%)
Transcatheter aortic valve implantation (TAVI)	673	67	267 (40%)	11 (2%)
Bronchial thermoplasty	651	65	73 (11%)	15 (2%)
Digital tomosynthesis	500	50	166 (33%)	12 (2%)
Fecal transplantation for *Clostridium difficile*	427	43	149 (35%)	22 (5%)
Intragastric balloon	226	23	104 (46%)	26 (12%)

Counts in the “articles identified” column add to 10 more than the 18,281 article total because 10 articles had been identified for possible inclusion in 2 projects

### Prioritization accuracy: EPPI-Reviewer and Abstrackr compared to traditional screening

**Fig. 3:**
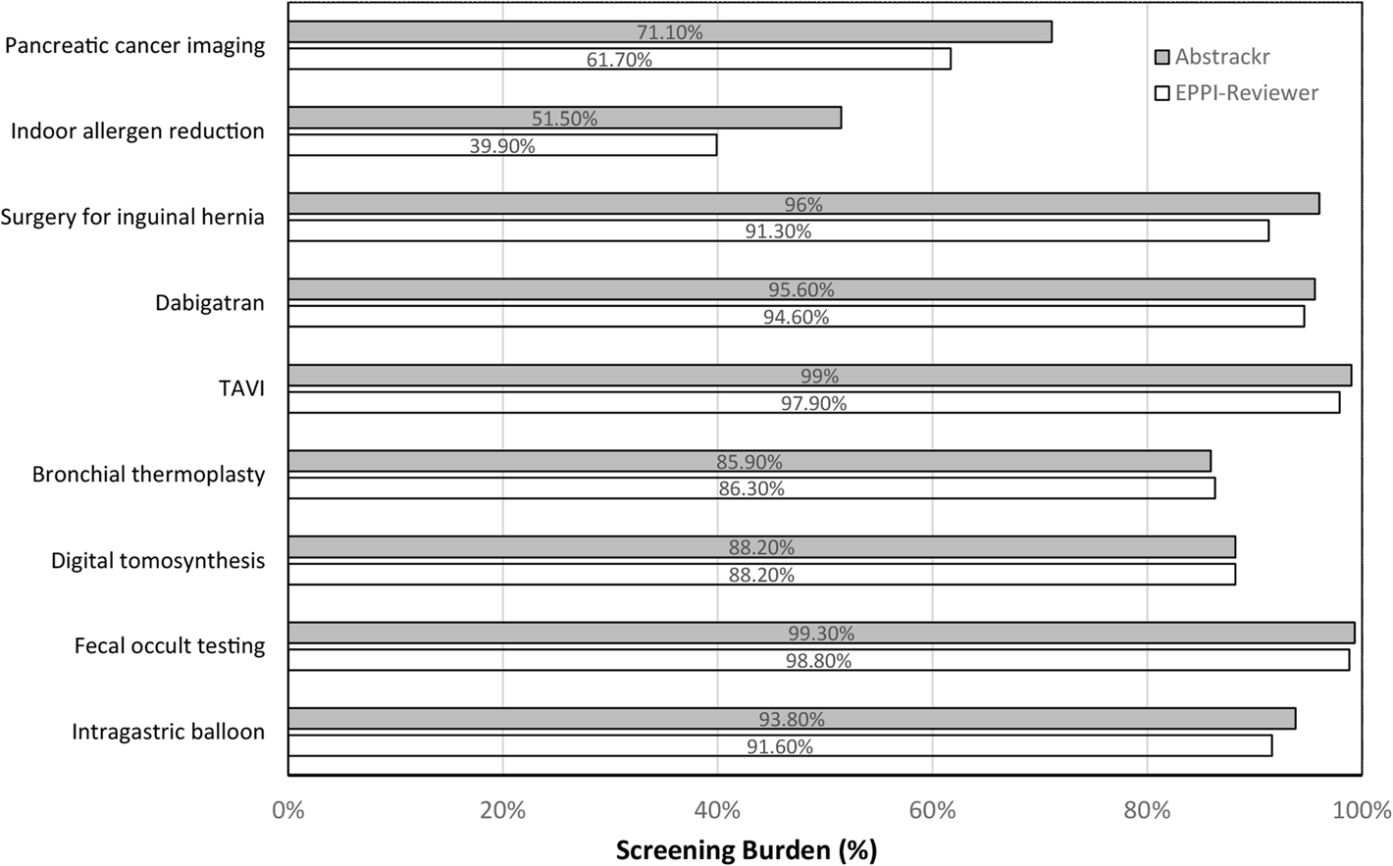
Prioritization accuracy for Task 1 (inclusion for full-text screening). This figure shows the percentage of articles screened by each tool to reach 100% of all articles ordered for full-text screening. Smaller bars indicate higher prioritization accuracy (as fewer articles had to be screened in order to achieve 100% sensitivity). The comparative performance of the two tools for each evidence report is displayed in gray (Abstrackr) and white (EPPI-Reviewer)

**Fig. 4:**
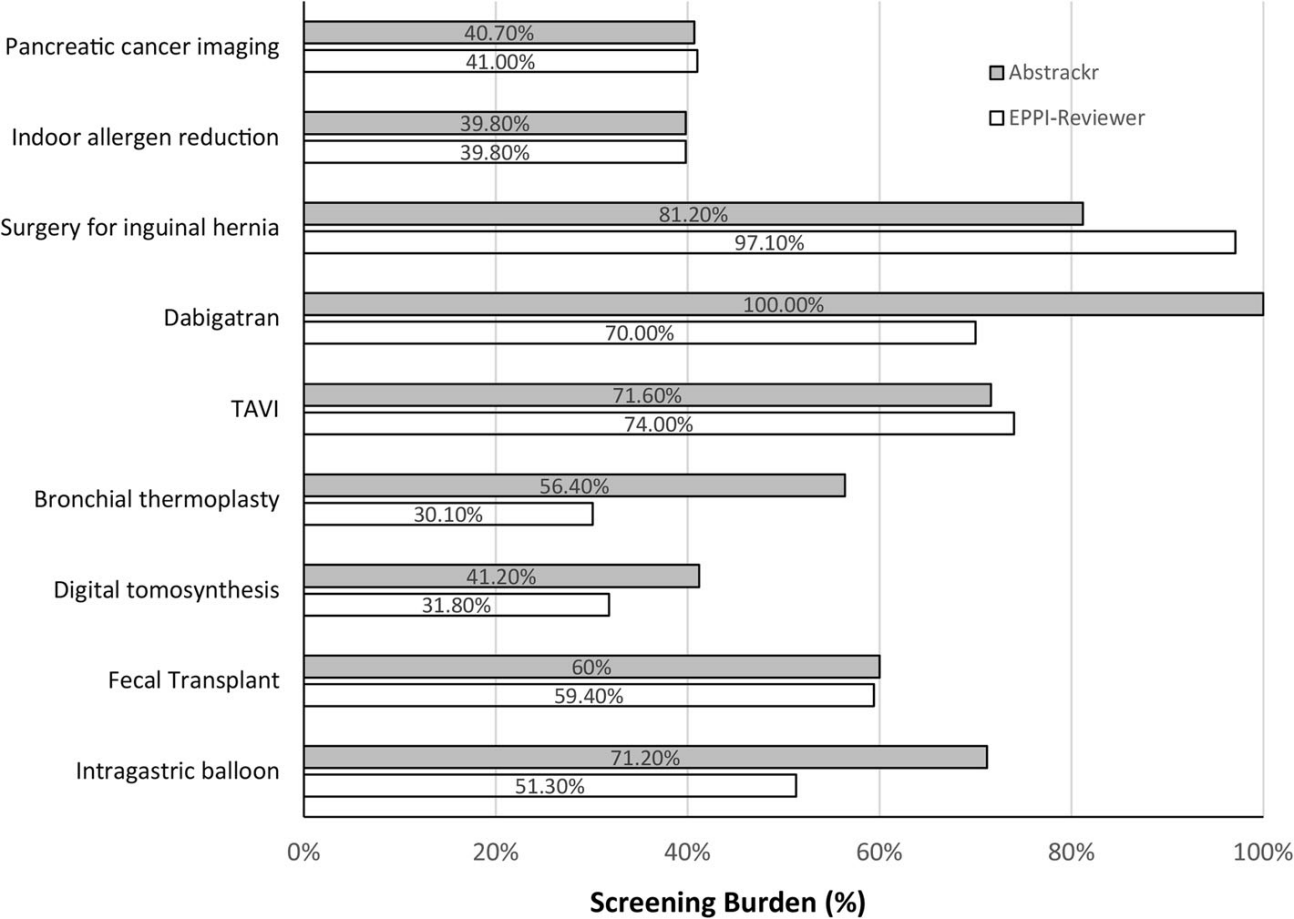
Prioritization accuracy for Task 2 (final inclusion). This figure shows the percentage of articles screened by each tool to reach 100% of all articles included in the final report. Smaller bars indicate higher prioritization accuracy (as fewer articles had to be screened in order to achieve 100% sensitivity). The comparative performance of the two tools for each evidence report is displayed in gray (Abstrackr) and white (EPPI-Reviewer)

The impact of EPPI-Reviewer and Abstrackr on the amount of screening necessary for these tasks is shown in Figs. 3 and 4 below. For Task 1 (identifying citations for full-text review), both tools demonstrated potential for large reductions in screening for 2 of 3 large reports. In fact, for the indoor allergen reduction report, nearly all relevant citations were identified by the time 50% of citations had been screened (screening burden of 39.9% and 51.5% for EPPI-Reviewer and Abstrackr, respectively). Both tools also reduced screening for the third large report (surgical options for inguinal hernia) as well, but these reductions were far smaller (< 10%). Both tools were much less accurate in prioritization for smaller reports, particularly in Task 1, likely due to smaller training sets.

To place the results in Fig. 3 into context, we calculated the potential number of abstracts that users could be “saved” from having to screen by using each tool (Table 3). For example, the table shows that for the pancreatic cancer imaging report, Abstrackr could have “saved” 2612 abstracts from needing to be screened. (This is the 28.9% of abstracts that were prioritized lower than the last ordered article (100% minus 71.1% that appears at the top of Fig. 3)). Overall, the numbers in Table 3 indicate substantial savings for the two largest reports, but modest savings for the other 7 reports.

**Table 3:** Abstract screenings saved

Report	Number of abstract screenings potentially saved by Abstrackr	Number of abstract screenings potentially saved by EPPI-Reviewer
***Large reports***		
Pancreatic cancer imaging (*N* = 9038)	2612	3462
Indoor allergen reduction (*N* = 3181)	1540	1912
Surgical procedures for inguinal hernia (*N* = 2706)	108	235
***Small reports***		
Dabigatran (*N* = 889)	39	48
Transcatheter aortic valve implantation (TAVI) (*N* = 673)	7	14
Bronchial thermoplasty (*N* = 651)	92	89
Digital tomosynthesis (*N* = 500)	59	59
Fecal transplantation for *Clostridium difficile* (*N* = 427)	3	5
Intragastric balloon (*N* = 226)	14	19

As expected in Task 2 (training the tool using studies ultimately included), both tools identified included studies faster (i.e., earlier) compared to Task 1 (full-text-review decisions). For instance, for 2 of 3 large reports all studies included in the final report were identified by the time 41% of citations had been screened. Similar to Task 1, both tools performed less well for the inguinal hernia report. Notably, both tools also performed well on the smaller reports, particularly EPPI-Reviewer, which reduced screening to < 60% for 4 of 6 small reports.

### Prioritization accuracy: EPPI-Reviewer vs. Abstrackr

**Fig. 5:**
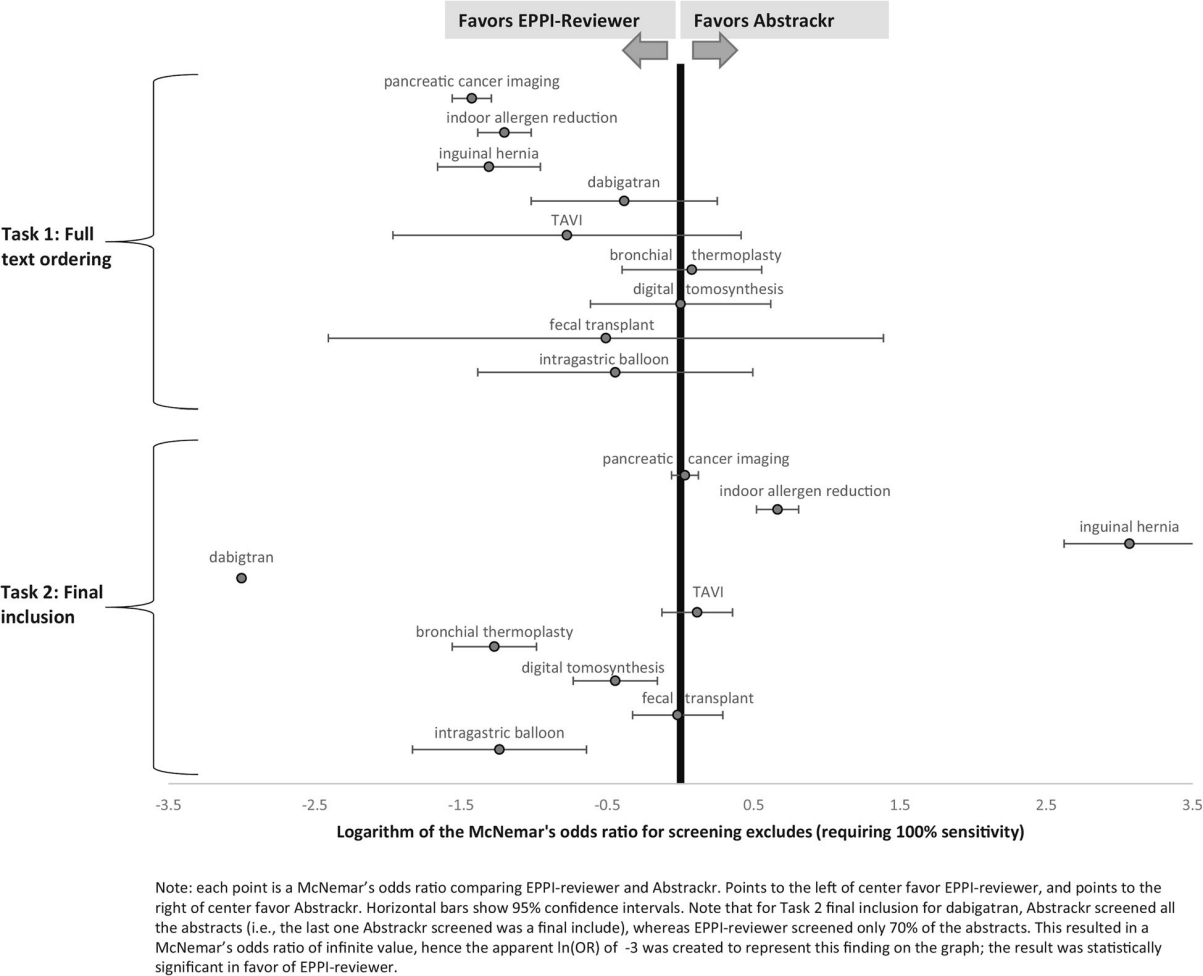
Statistical comparisons of screening burden. Each point is a McNemar’s odds ratio comparing EPPI-Reviewer and Abstrackr. Points to the left of center favor EPPI-Reviewer, and points to the right of center favor Abstrackr. Horizontal bars show 95% confidence intervals. Note that for dabigatran, in Task 2 (final inclusion), Abstrackr required all abstracts to be screened before reaching the last included study, while EPPI-Reviewer only required 70% of abstracts screened to identify all included studies. This resulted in a McNemar’s odds ratio of infinite value, hence the apparent ln (OR) of − 3 was created to represent this finding on the graph; the result was statistically significant in favor of EPPI-Reviewer

We compared performance of the two tools using McNemar’s odds ratio. In Task 1, EPPI-Reviewer performed better than Abstrackr for all 3 large reports, consistently identifying relevant studies for full text ordering faster than Abstrackr (see Fig. 5, where for the top 3 datapoints, confidence intervals are fully to the left of a null effect). For example, for Task 1 for pancreatic cancer imaging, EPPI-Reviewer demonstrated a potential reduction in screening burden of nearly 10% compared to Abstrackr, identifying all abstracts ordered for full text 868 citations earlier. For indoor allergens and surgical interventions for inguinal hernia, EPPI-Reviewer provided reductions of 12% and 5% translating to 369 and 127 citations, respectively. EPPI-Reviewer also appeared to perform better for several of the smaller reports, although those results did not reach statistical significance.

However, for Task 2 (final inclusion predictions) results were mixed: Abstrackr performed better for reviews on indoor allergens and surgical interventions for inguinal hernia, but EPPI-Reviewer performed better for dabigatran, bronchial thermoplasty, digital tomosynthesis, and intragastric balloon (all statistically significant).

### Sensitivity at progressive screening thresholds

**Fig. 6:**
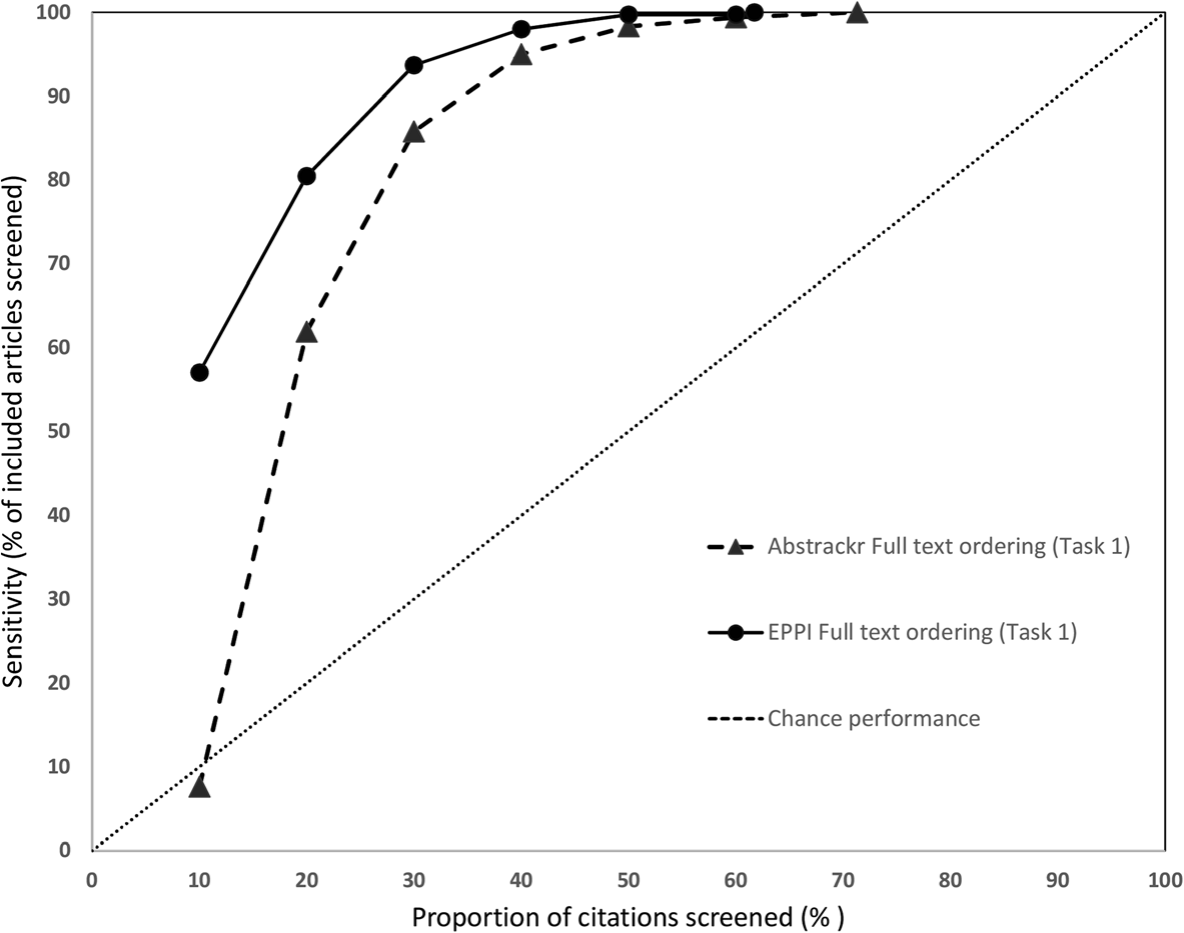
Sensitivity at various thresholds for pancreatic cancer imaging (Task 1). This figure shows the proportion of included articles that were screened at each 10% increment. Performance for both Abstrackr (dotted lines with triangles) and EPPI-Reviewer (solid lines with circles) is plotted. Chance performance is shown by the 45 degree line. As the *y*-axis plots sensitivity, curves closer to the top left of the graph indicate faster learning

**Fig. 7:**
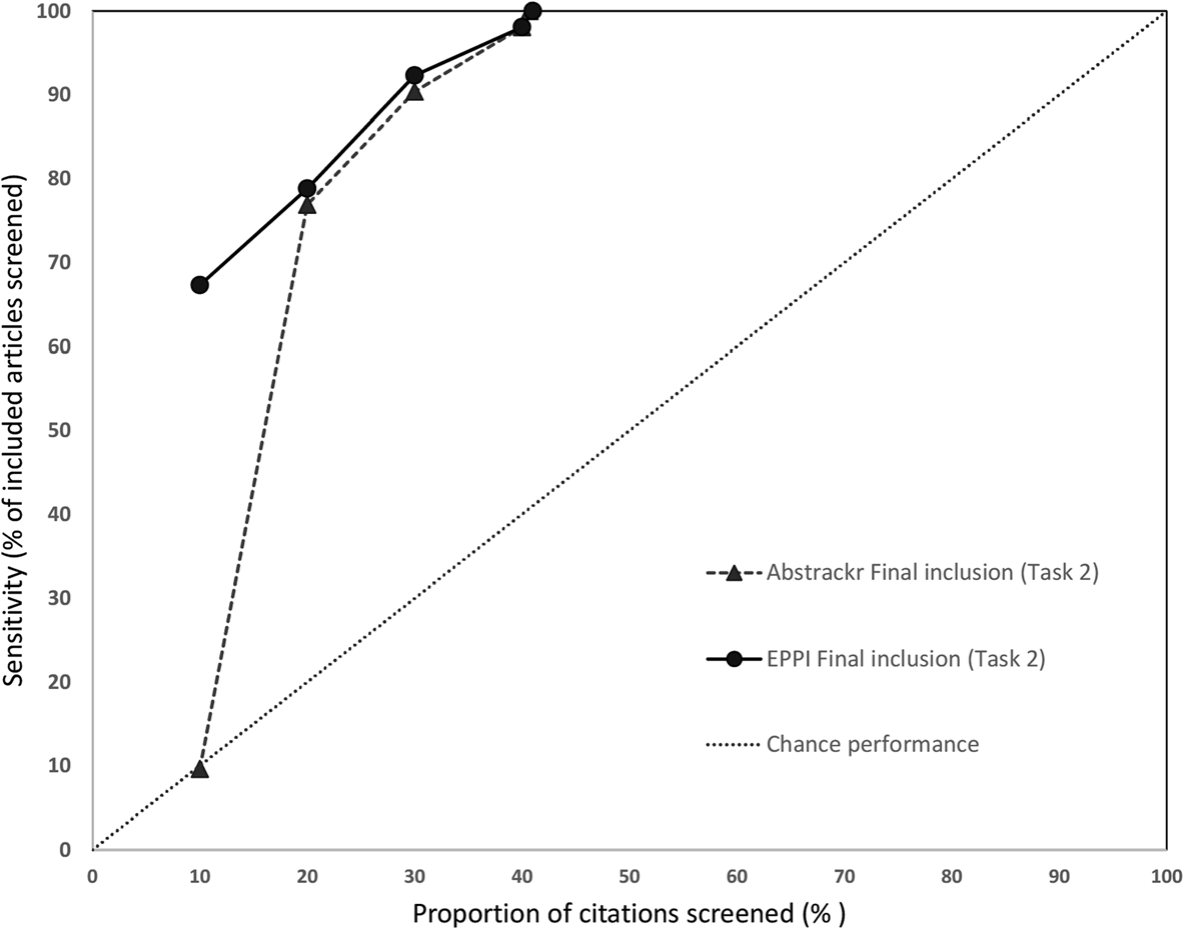
Sensivity at various thresholds for pancreatic cancer imaging (Task 2). This figure shows the proportion of included articles that were screened at each 10% increment. Performance for both Abstrackr (dotted lines with triangles) and EPPI-Reviewer (solid lines with circles) is plotted. Chance performance is shown by the 45 degree line. As the *y*-axis plots sensitivity, curves closer to the top left of the graph indicate faster learning

**Fig. 8:**
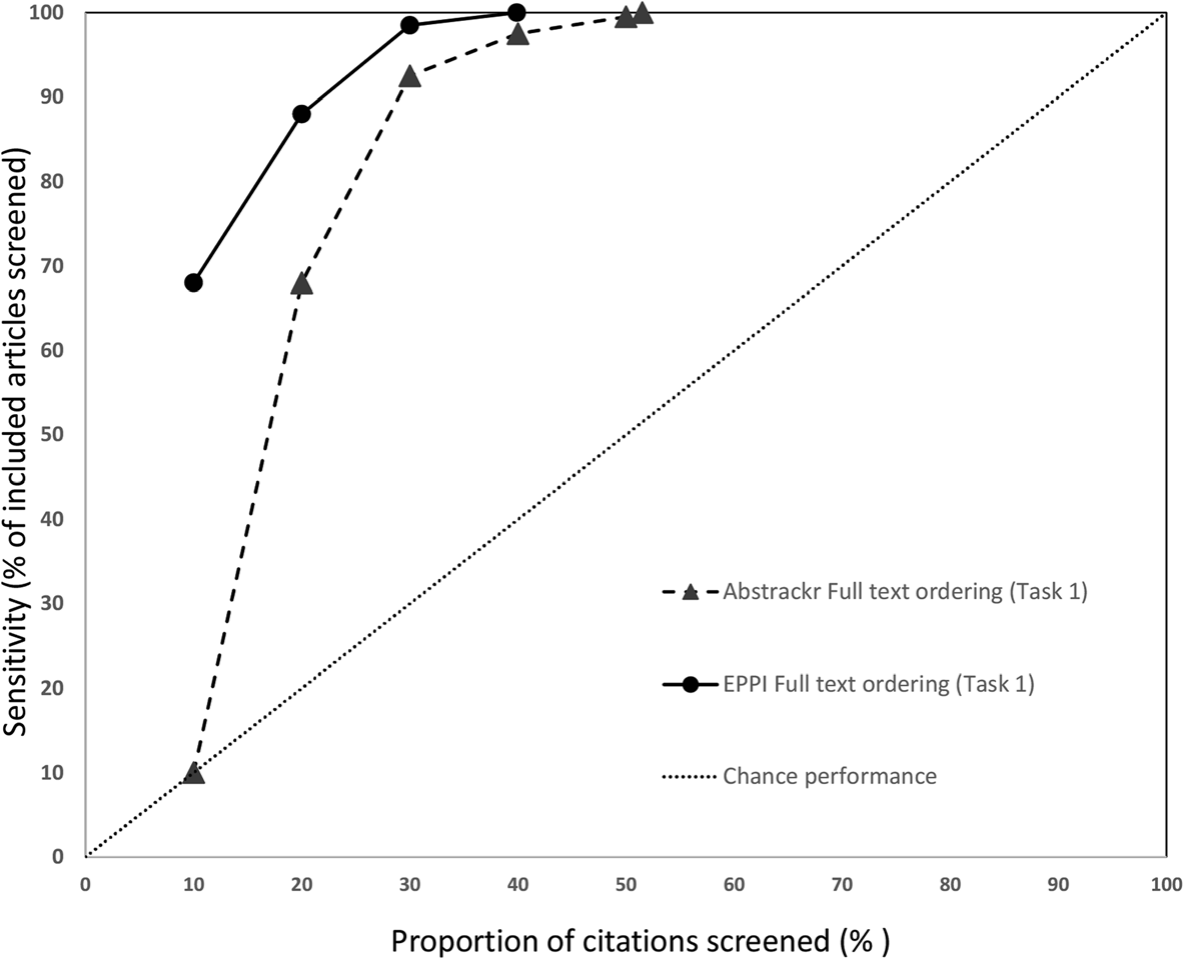
Sensitivity at various thresholds for indoor allergen reduction (Task 1). This figure shows the proportion of included articles that were screened at each 10% increment. Performance for both Abstrackr (dotted lines with triangles) and EPPI-Reviewer (solid lines with circles) is plotted. Chance performance is shown by the 45 degree line. As the *y*-axis plots sensitivity, curves closer to the top left of the graph indicate faster learning

We also assessed how many relevant citations had been identified at successive 10% intervals for each screening tool. Data for the 3 large reports are shown in Fig. 6 (pancreatic cancer), Fig. 7 (indoor allergens), and Fig. 8 (inguinal hernia) (we did not produce these graphs for the small reports, due to small Ns). In these figures, the horizontal axis represents the percentage of all articles screened (in 10% increments), while the vertical axis plots the sensitivity (defined as the percentage of included articles screened at that point).

As each tool was trained with 10% of citations and Abstrackr only updates daily, at 10% of citations screened, EPPI-Reviewer consistently outperformed Abstrackr. This was expected, as Abstrackr did not have the opportunity to re-order citations until 10% had been screened; thus, the order at 10% screened simply reflects a random 10% sample. By contrast, for EPPI-Reviewer, after 10 random citations were screened as “training,” we engaged the screening prioritization functionality, allowing EPPI-Reviewer to iteratively reorder citations as soon as sufficient data for prediction was acquired. EPPI-Reviewer’s edge over Abstrackr at 10% of citations screened was expected, but nevertheless demonstrates the pragmatic advantages to EPPI-Reviewer’s iterative approach which allows identifying relevant articles earlier.

**Fig. 9:**
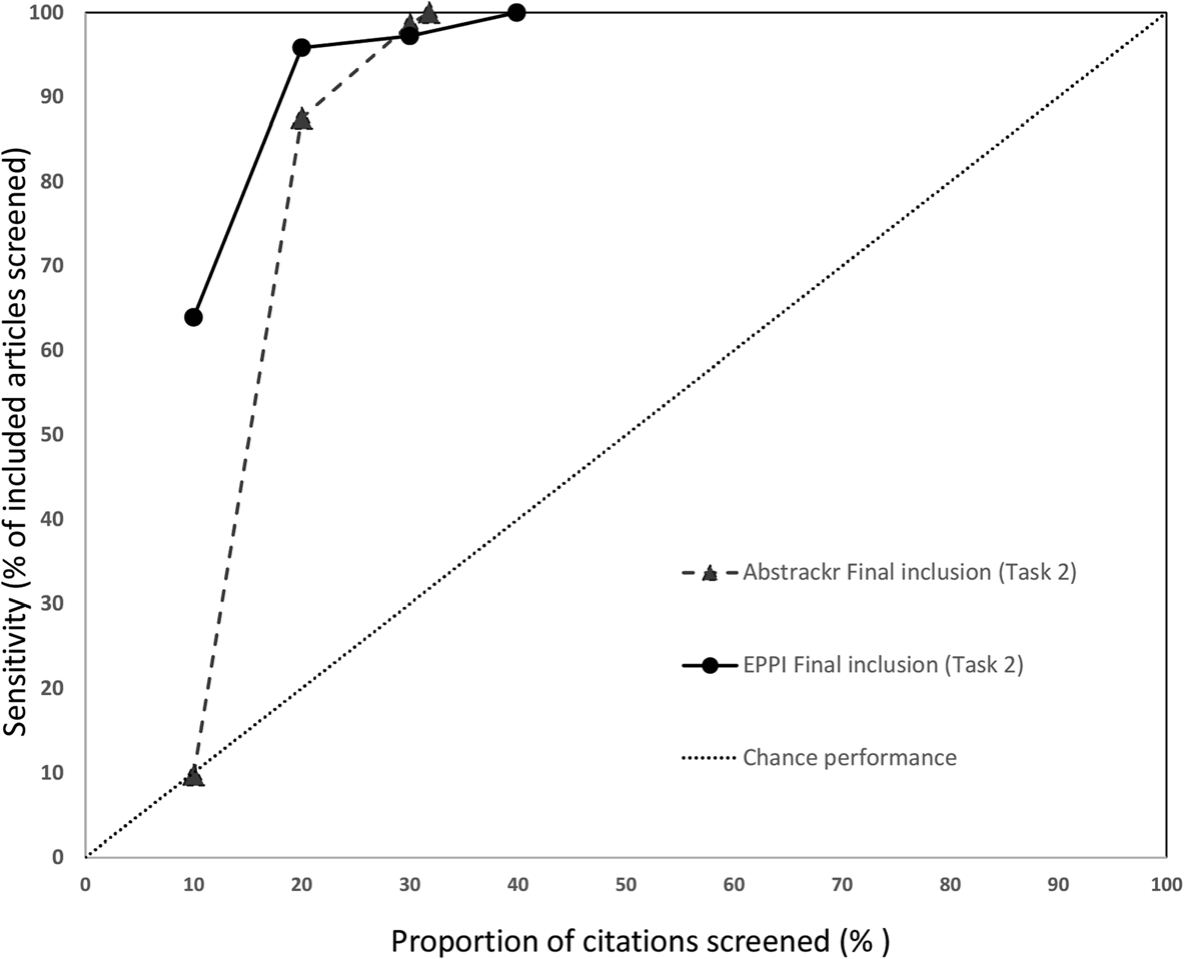
Sensitivy at various thresholds for indoor allergen reduction (Task 2). This figure shows the proportion of included articles that were screened at each 10% increment. Performance for both Abstrackr (dotted lines with triangles) and EPPI-Reviewer (solid lines with circles) is plotted. Chance performance is shown by the 45 degree line. As the *y*-axis plots sensitivity, curves closer to the top left of the graph indicate faster learning

Both tools learned quickly for 2 of 3 large reviews (pancreatic cancer imaging and indoor allergen reduction). For pancreatic cancer, at only 30% citations screened, both tools had already identified ≥ 85% of all relevant studies in both tasks. By 40% screening, both tools had screened 98.1% of studies included in the final report (Task 2). For indoor allergen reduction, performance was even more impressive: at only 30% screening, Abstrackr had screened 98.6% of the final included studies, and EPPI-Reviewer had screened 97.2%. Perfect sensitivity (100%) was achieved by screening only 31.8% of citations (Abstrackr) and 39.9% (EPPI-Reviewer) (Fig. 9).

**Fig. 10:**
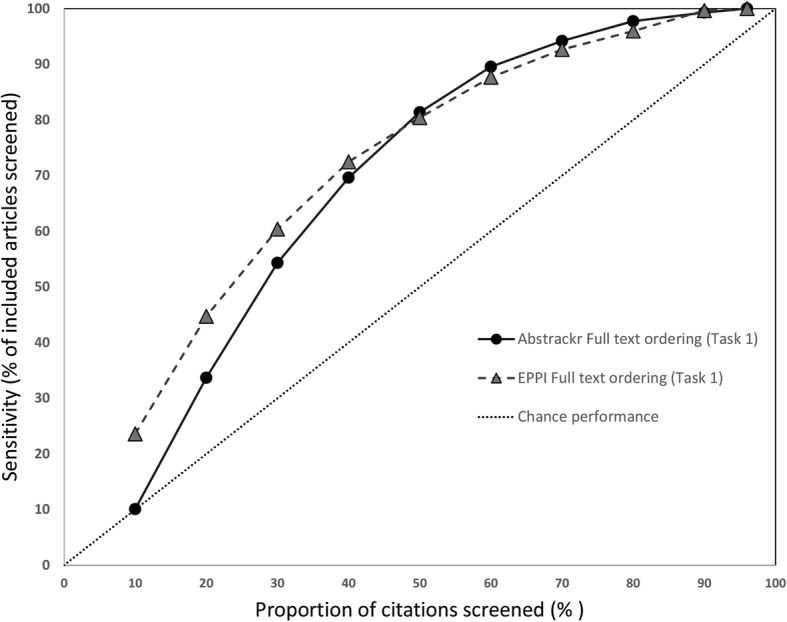
Sensitivity at various thresholds, surgical interventions for inguinal hernia (Task 1). This figure shows the proportion of included articles that were screened at each 10% increment. Performance for both Abstrackr (dotted lines with triangles) and EPPI-Reviewer (solid lines with circles) is plotted. Chance performance is shown by the 45 degree line. As the *y*-axis plots sensitivity, curves closer to the top left of the graph indicate faster learning

**Fig. 11:**
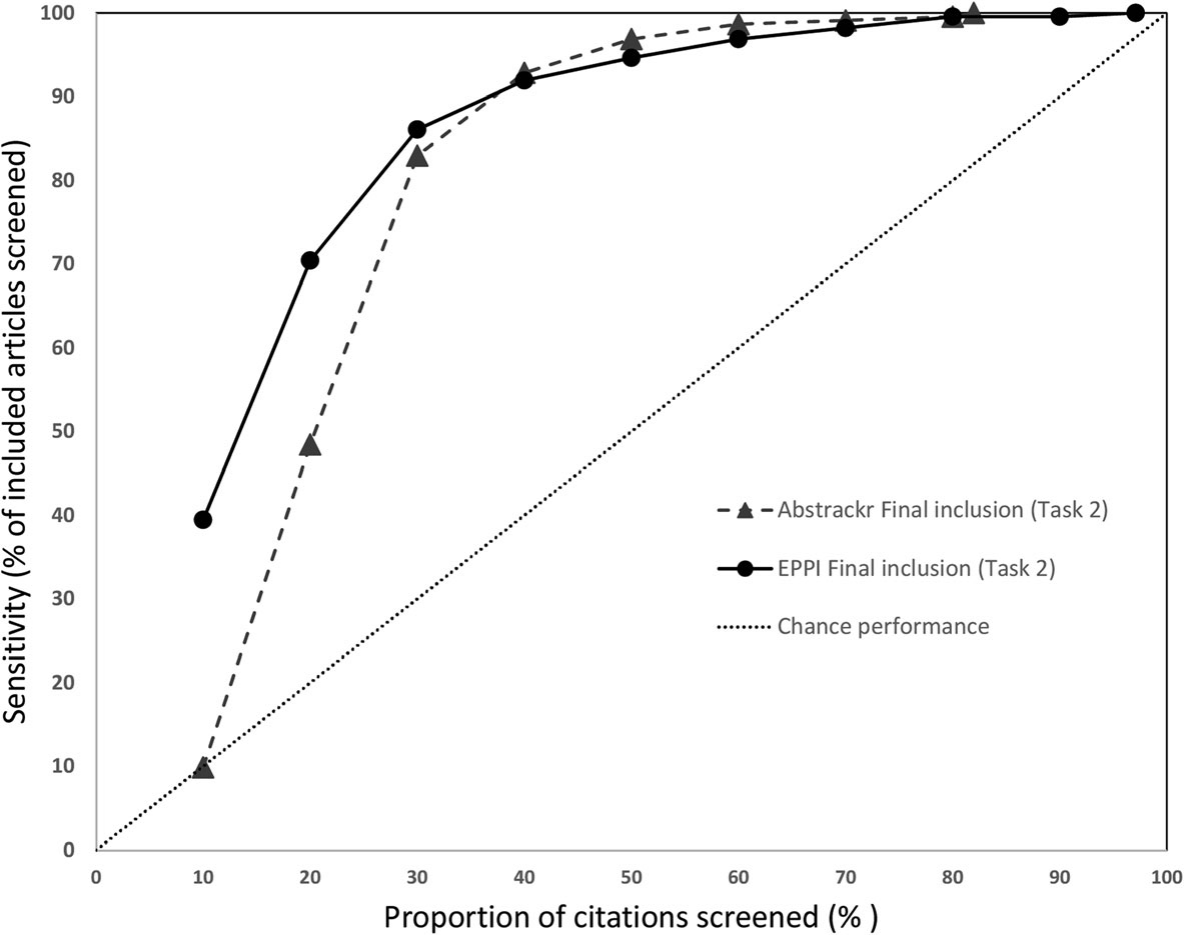
Sensivity at various thresholds, surgical interventions for inguinal hernia (Task 2). This figure shows the proportion of included articles that were screened at each 10% increment. Performance for both Abstrackr (dotted lines with triangles) and EPPI-Reviewer (solid lines with circles) is plotted. Chance performance is shown by the 45 degree line. As the *y*-axis plots sensitivity, curves closer to the top left of the graph indicate faster learning

Performance was poorer for the third large review, inguinal hernia (Figs. 10 and 11). Although a significant proportion of citations included in the report were reached by 30% of citations screened (83% for Abstrackr, 86% for EPPI-Reviewer), very high sensitivity (99% for Abstrackr, 98% for EPPI-Reviewer) was not reached until 70% of citations had been screened. For both tools, 21 relevant citations were not screened until the bottom 50% of all articles. Thus, although performing substantively better than chance, both tools performed less well for this project compared to the other 2 large projects.

## Discussion

Our study found both Abstrackr and EPPI-Reviewer have the potential to reduce screening burden. Specifically, for large reports, potential reductions in screening burden were 4 to 49% (Abstrackr) and 9 to 60% (EPPI-Reviewer). Our findings are consistent with other studies which have found Abstrackr to offer potential gains [7, 19, 21]. However, to our knowledge, our study is the first to assess EPPI-Reviewer. Overall, compared to Abstrackr, we found that EPPI-Reviewer offered similar prioritization accuracy. In 2 of 3 large evidence reports, both tools performed well for both Tasks 1 and 2.

Performance for the third large report (surgical interventions for inguinal hernia) was less impressive for both tools: in Task 1, 91.3% and 96% of citations required screening (for EPPI-Reviewer and Abstrackr respectively) before all citations ordered for full text were screened. One possibility for this poorer performance could be that citations not identified until late in the screening process represent studies that were somewhat “off topic,” but were included for further review as part of a cautious approach. In this scenario, one might expect many of these studies to ultimately be excluded from the final report. In this case, the prediction algorithm might be unfairly penalized for designating these citations low priority for full text ordering (e.g., correctly assessing the potential for inclusion as very low). However, our review of studies identified very late for Task 1 (full text ordering) suggests some citations falling in this “tail” *were* ultimately included in the final report. After 70% of citations had been screened, both citation tools had not yet identified 4 (of 223 total) studies that would be included in the final report.

These studies identified late in screening may have been harder for Abstrackr/EPPI-Reviewer to identify because they differed in key ways from studies identified earlier in the process. Of 223 studies included, 62 (reporting 32 unique studies) addressed a key question on the learning curve for laparoscopic hernia repair. Inclusion criteria for this key question differed from other questions in the report; specifically, many single-arm studies excluded from consideration for other key questions were allowed for this key question. Of 62 publications included for this key question, 57 were in the bottom 50% of priority for either Abstrackr or EPPI-Reviewer (or both). This suggests the other 161 included publications, many of which described randomized trials, may have influenced algorithms to prioritize only randomized trials.

If machine learning algorithms are focused on clinical content or study design, studies included or excluded based on a *non-clinical* study characteristic (e.g., publication date or study size) would likely reduce predictive power. One potential strategy for minimizing the impact of such factors in the future would be to isolate key questions with distinctive populations or inclusion criteria by separating them from other key questions (i.e., training algorithms for these questions separately).

Interestingly, the original search for surgical interventions for inguinal hernia was distinctly more challenging, perhaps due to the fact that it is a procedure-based topic not well-indexed with controlled vocabulary terms. It is possible that the same factors creating challenges for crafting a search with adequate sensitivity and specificity could also pose problems for machine learning algorithms.

### Small reports

For smaller reports, neither tool offered significant gains for Task 1 (predicting full text ordering). However, for Task 2 (which trained tools using only articles included in the final report) both tools performed surprisingly well, particularly EPPI-Reviewer. In 4 of 6 small reports, EPPI reviewer identified all included citations earlier than Abstrackr, offering reductions in screening burden of 30 to 70% (see Fig. 5). This suggests that even for small reports, tools like EPPI-Reviewer can offer efficiency gains, particularly for playing a confirmatory role (e.g., checking for missed citations at the end of a review) or updating reviews, both situations in which a high-quality training set is available.

### Encouraging wider adoption

Despite numerous tools for automation, adoption of these tools has lagged [8, 22]. Reasons for slower uptake may include pragmatic challenges from incorporating tools into current workflow, and lack of trust by the systematic review community [22]. Many studies evaluating these tools have assessed automatic classification functionality, in which screening continued until the tool predicts whether studies should be included or not, and remaining citations are not screened [19, 21]. However, existing uncertainties regarding how to ensure tools like Abstrackr and EPPI-Reviewer perform at their best suggest that perhaps utilizing these tools for screening prioritization remains the safest choice for those concerned about missing any relevant citations. If a “trust gap” is impeding adoption, perhaps encouraging initial use of these tools for screening prioritization, while accumulating new evidence for specific contexts in which automatic classification may safely be used is the way forward.

### Special considerations for use

Using screening prioritization tools may require careful consideration of factors which could potentially reduce predictive performance. For instance, as previously noted, we carefully removed all duplicates from the citation set. If one study is included, but a second study is excluded as duplicate (i.e., not due to irrelevant content), this could potentially confuse an algorithm learning on content alone. Similarly, excluding studies based on non-clinical characteristics such as publication year, country, or sample size could similarly confuse the prediction algorithm. For example, if a study evaluated an intervention of interest, but was excluded solely based on small sample size, the predictive algorithm might mistakenly conclude the intervention was irrelevant.

Secondary publications may also pose a challenge: if one study population appears in multiple publications, authors may elect to include the “primary” original study, while excluding secondary publications (additional publications from the same study). Reviewers would need to be cognizant of the possibility of sending mixed messages to the machine learning algorithm. To maximize benefits from these tools, reviewers would either need to specify inclusion of all secondary publications or “clean” the citation set prior to training the citation screening tool.

### Limitations

Our study has important limitations. Performance of Abstrackr and EPPI-Reviewer may significantly vary depending on composition of the initial training set. We trained Abstrackr using a random 10% of citations. If studies in this training set were not representative, the predictive algorithm’s “first impressions” may have been skewed, affecting performance (e.g., repeating the study with a different random 10% training set could have produced different results). Similarly, we used a random 10 citations to train EPPI-Reviewer; using a different 10 studies could produce different results. Also, notably, both tools offer features which could improve performance in “real life” use (e.g., identification of key words and automatic classification) which we chose not use, in order to facilitate comparison across tools.

Similarly, we assessed performance at intervals of 10% citations screened rather than counts of numbers of studies (e.g., every 100 citations). We chose 10% intervals to capture incremental improvement with progression of screening, but also to accommodate pragmatic considerations. For instance, as Abstrackr updates the order of citations only once a day, to complete screening of > 9000 citations for the pancreatic cancer imaging report in 100 citation increments would have required 90 days. However, we recognize a 10% increment translates to significant differences in actual number of citations screened across reports (e.g., 904 citations for the pancreatic imaging report compared to only 271 citations for inguinal hernia) and results may have differed had we analyzed results by number of citations screened alone.

Despite the potential for significant reductions in screening burden (particularly for large reports with high numbers of citations) a fundamental barrier to wider uptake is the inability to know in advance whether tool performance is excellent (e.g., pancreatic cancer imaging and indoor allergens) or less good (e.g., inguinal hernia). For example, for the pancreatic imaging report, EPPI-Reviewer identified all citations ordered for full-text review after only 62% of citations had been screened. However, in “real life” use, systematic reviewers cannot simply stop screening at this point (to avoid screening the remaining 3400 citations). Prospectively, it remains impossible to discern whether the predictive performance will be “good” or “bad.” Thus, our data on “potential” reductions in screening burden may not translate into actual reductions for real-time use.

Nevertheless, our findings suggest that a conservative approach could expect to identify nearly all citations for inclusion after screening 70–80% of citations using these tools. A more daring approach would be to stop screening after 50% of citations have been screened, which our study suggests would still identify roughly 95% of included studies. More work is needed to evaluate the tradeoffs associated with truncating screening early (e.g., at 50%) to assess to what extent these “missed” studies impact the report’s conclusions. Although this technology is in the early stages of adoption, as these tools become more commonplace and integrated into routine SR development, some of these factors may change. Training sets based on initial versions of SRs could facilitate highly desirable living SRs where new citations are considered for relevancy in a predetermined ongoing process. Libraries of training sets (along with key questions they were designed to answer) potentially could streamline prioritization for related new reviews.

## Conclusions

Both EPPI-Reviewer and Abstrackr performed well for predicting relevant citations, both for full-text review, and for final inclusion. Our findings suggest these tools are safe for use in confirmatory roles (e.g., to double-check if relevant citations have been missed) or for use in ongoing systematic reviews, as many have suggested. As expected, for new review development, these tools were most beneficial for larger citation sets (with ≥ 2500 citations). While these tools can expedite workflow efficiency by identifying relevant citations earlier, more work is needed to determine the impact of studies that may be missed if screening is truncated below a particular threshold. Future research should also assess the risks and tradeoffs associated with using these tools prospectively.

### Significance

Data from this project may inform decisions on how to shorten the article screening process. Such decisions might arise in the context of a rapid review, a review update, or general efficiency gains.

## Data Availability

The datasets generated and analyzed during the current study are not publicly available as other research projects are ongoing but are available from the corresponding author on reasonable request.
